# Massive Nasal Arterio-Venous Malformation (AVM) Excision and Reconstruction with Expanded Forehead Flap: A Case Report

**Published:** 2017-01

**Authors:** Ghasem Ali Khorasani, Siamak Rakei, Hooman Riazi

**Affiliations:** 1 Tehran University Medical Sciences, Tehran, Iran;; 2 Arya Hospital, Tehran, Iran

**Keywords:** Arterio-venous malformations, Vascular lesion, Embolization

## Abstract

Nasal arterio-venous malformations (AVM) are uncommon lesions. We present a rare case of huge, long standing AVM in the nasal area which was treated by angioembolization, followed by surgical excision and forehead flap reconstruction.

## INTRODUCTION

Vascular lesions of the head and neck can cause cosmetic issues for the patient and some of them may lead to serious, life-threatening bleeding. Vascular lesions are divided into “vascular tumors” (which have increased endothelial cell turnover) and “vascular malformations” (which do not have increased endothelial cell turnover).^1^ Hemangiomas are the most common vascular tumors. They typically present during infancy and are more prevalent in females.^2^ Hemangiomas are the most common tumors of the head and neck in infancy and childhood which are in differential diagnosis with vascular malformation. Their clinical presentation is a vascular lesionin nose, ears or eyelids.^3^^,^^4^

These lesions usually resolve over time. Concomitant hemangiomas can be seen in other organs, most commonly in liver, especially in case of multiple cutaneous hemangiomas.^5^ Vascular malformations are sub-classified into capillary, venous, arterial and lymphatic, based on the tissue type. They are also divided into two categories: low-flow and high-flow lesions. Capillary, venous and lymphatic malformations are “low-flow” lesions while arterial and arterio-venous malformations (AVM) are “high-flow” and are capable of severe hemorrhage with significant morbidity.^5^

## CASE REPORT

**Fig. 1:**
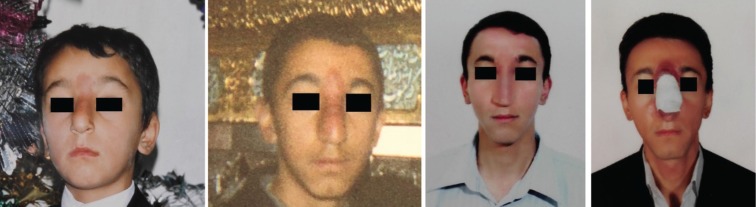
The lesion first appeared when the patient was 9 years old as a small, solitary, non-ulcerated, slow growing lesion

The patient was a 28-year-old man with a huge, necrotic, erythematous inflamed lesion on the glabella, nasal dorsum and paranasal area. On physical exam, the lesion was not tender or warm, did not have pulsation and the margins were vague. The patient first noticed the lesion when he was 9 years old. At the beginning, it had been an erythematous lesion in the nasal area which enlarged over time and used to bleed with patient’s manipulation (Figure 1). No concomitant vascular lesion or family history of arterio-venous malformation was found. The patient did not receive any treatment until 20 years of age, when he was visited in another center and undergone sclerotherapy by bleomycin, after which he says the lesion enlarged.

**Fig. 2:**
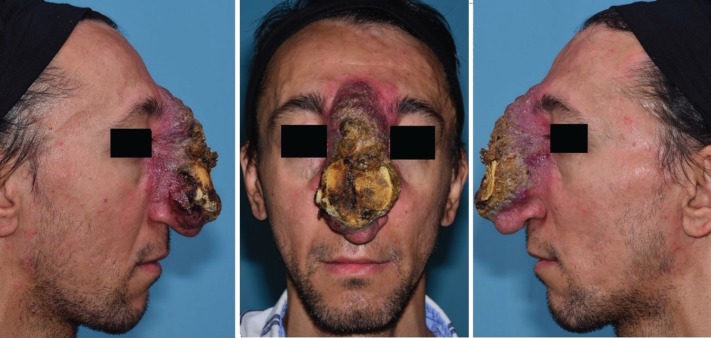
The patient at 28 years of age, when he was visited in our center. Staged-surgery was planned for the patient.

**Fig.3:**
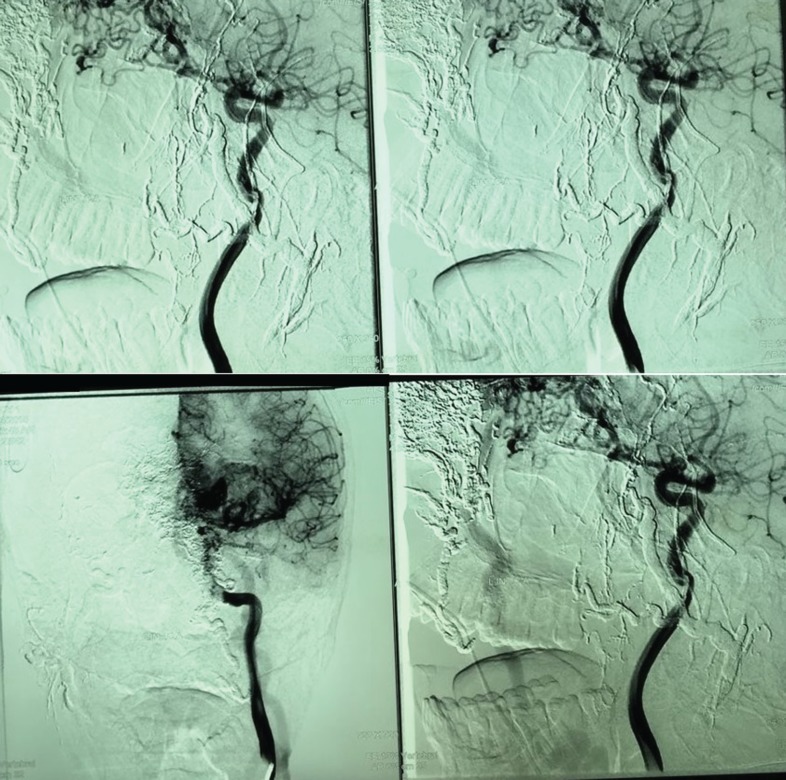
High flow AVM

At age 28, he was visited in our center (Figure 2). MRI and CT-scan was performed which revealed a soft tissue mass without bone or cartilage involvement. CT-angiography showed a high-flow vascular lesion. Color Doppler reported a vascular lesion with dilated vessels. Stage 1: A size 90 tissue expander was inserted in the forehead area. Supratrochlear artery was identified by color Doppler and included in the flap region. Expansion was done for 10 weeks until the desired size was achieved (Figure 3).

**Fig. 4:**
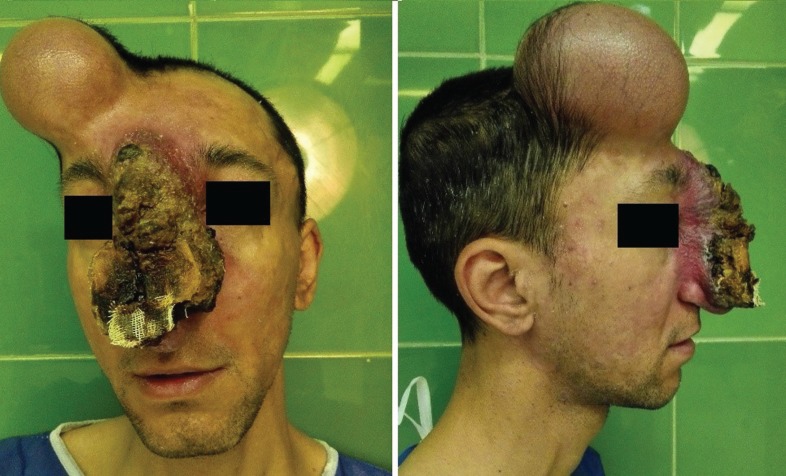
Tissue expander in fore head flap area

Stage 2: Embolization was done to reduce the size of the lesion and the risk of bleeding. However, no obvious change in the size, color or consistency of the lesion was noticed. Stage 3: After 48 hours the lesion was excised. Before excision, two rows of deep sutures were applied around the lesion to reduce the risk of bleeding. The feeding vessels of AVM were ligated and the lesion was completely excised off the bone and cartilage and the wound was left open (Figure 3 and 4).

**Fig .5:**
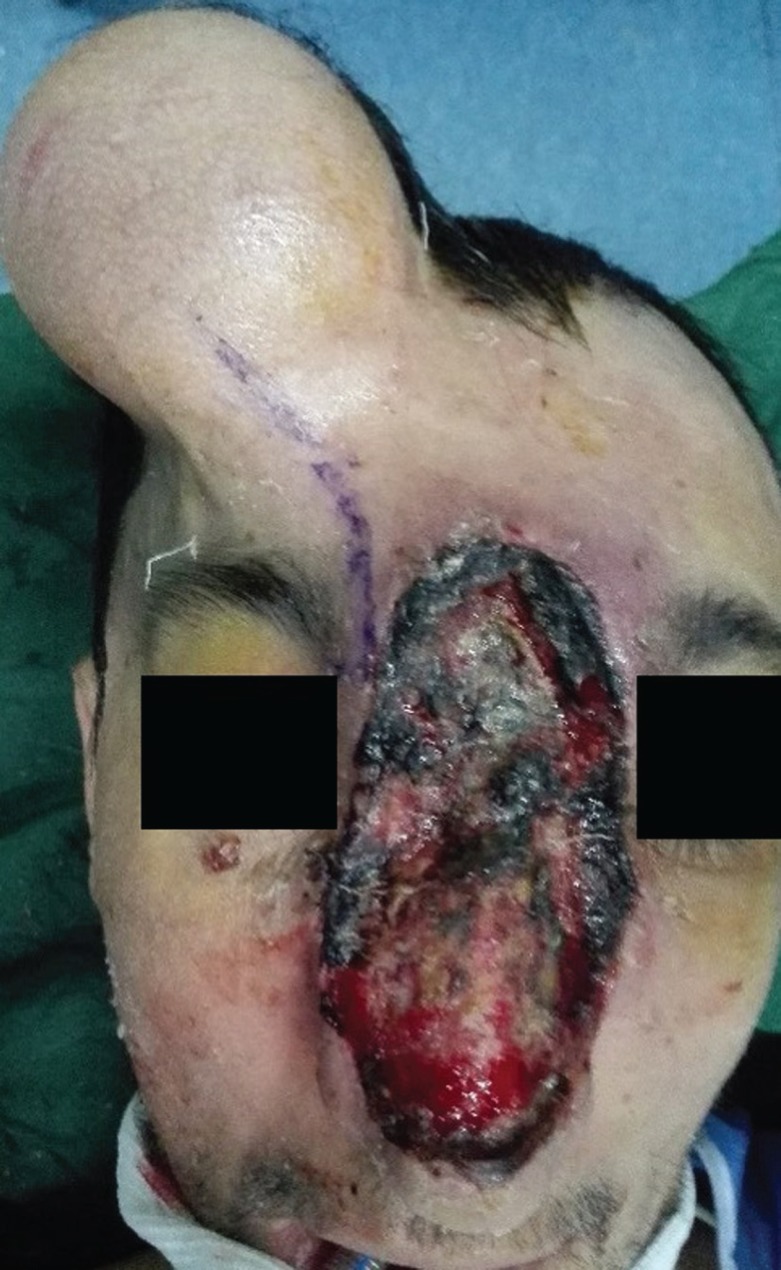
AVM excised

**Fig .6:**
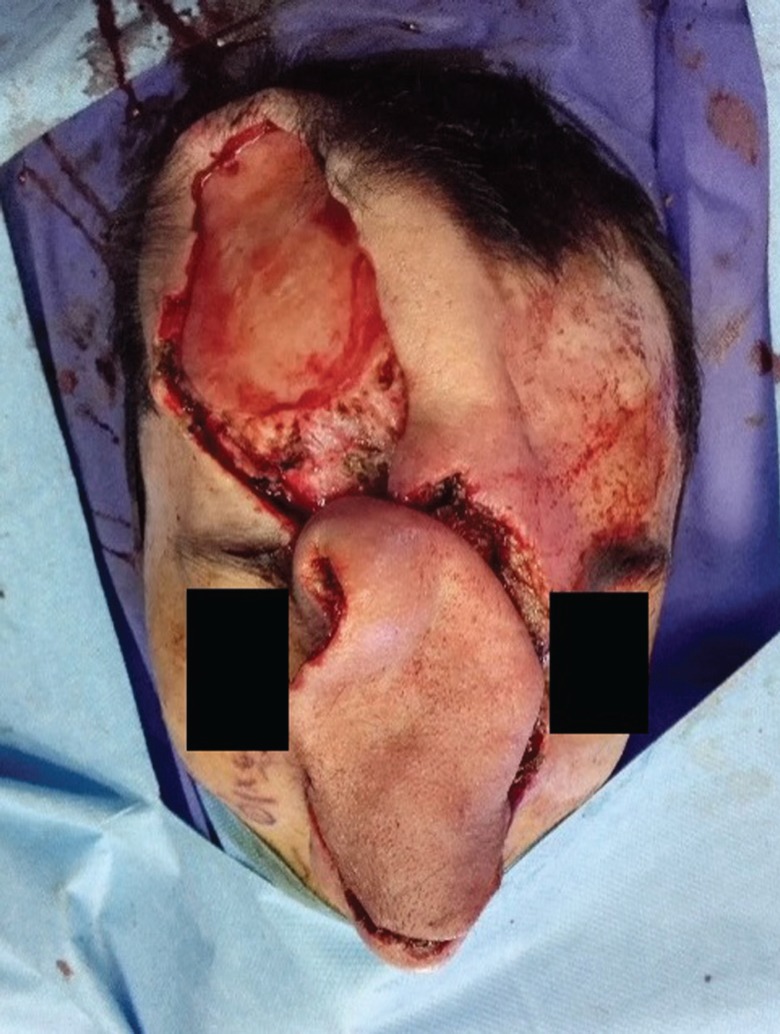
The forehead flap

**Fig .7:**
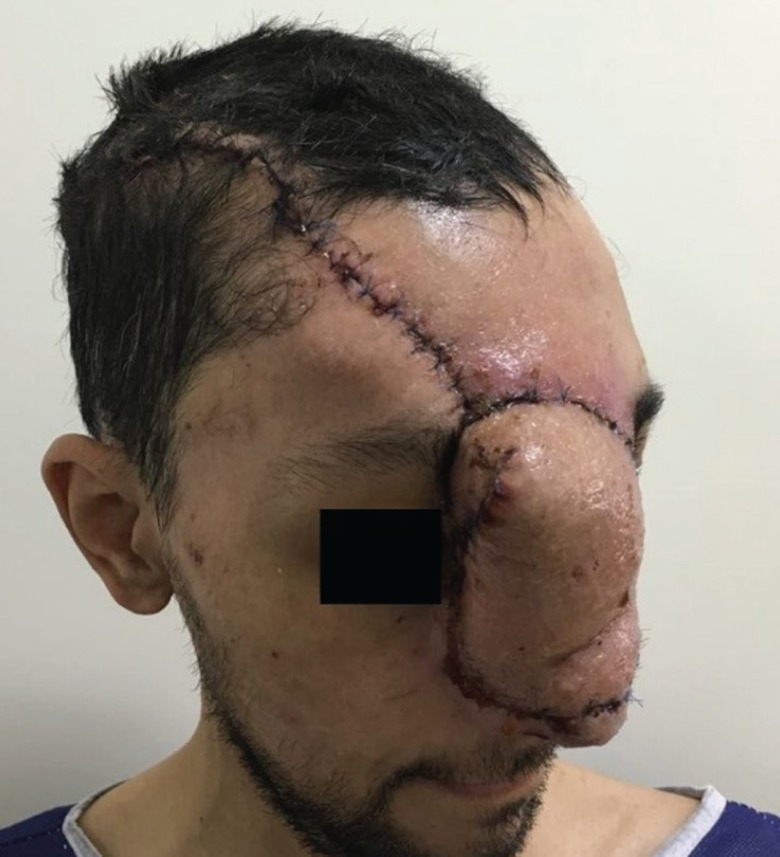
Three days after the operation

**Fig .8:**
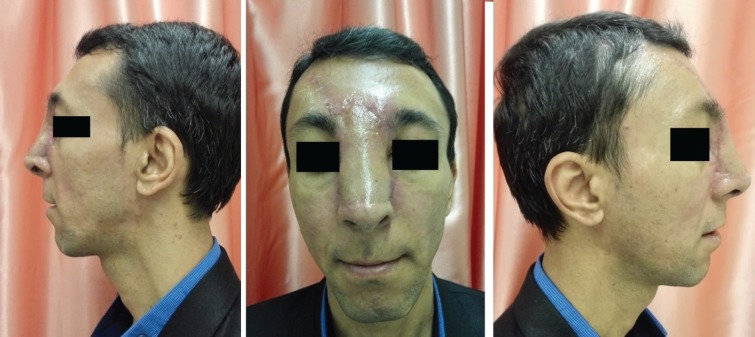
Six weeks after the operation

Stage 4: After another 48 hours the patient was taken to the operating room again. The wound was irrigated. An extended forehead flap on the base of supratrochlear artery was designed and rotated to cover the defect site. The donor site was closed primarily (Figure 5 and 6). The patient did not need transfusion during surgery or after it and he did not show any complication over 3 months post-operative follow up (Figure 7 and 8).

## DISCUSSION

AVMs are diagnosed by history and physical examination. They usually present as a pink-red cutaneous stain without a palpable trill or bruit. These patients are at risk of pain, ulceration or bleeding. They may also cause disfigurement and vital structure obstruction. Moreover, AVMs may eventually lead to high-output cardiac failure. Magnetic resonance imaging (MRI), CT-scan and Doppler are all used for vascular malformation evaluation, to document the lesion’s extension to the surrounding structures and to differentiate low-flow from high-flow lesions.^6^

There are different therapeutic methods for AVMs, including sclerotherapy, embolization, stereotactic radiation and surgery, which are used in combination.^7^ As we noticed in this patient, sclerotherapy alone was not effective. Selective embolization is usually used pre-operatively as an adjuvant therapy, to reduce bleeding and shrink the lesion, followed by surgical debulking.^8^ These lesions are usually excised macroscopically. Surgery is necessary to correct facial deformities.^9^^-^^12^

To conclude, a multi-disciplinary approach, including selective embolization followed by surgical excision and appropriate reconstruction is the best treatment for head and neck AVMs. In some cases (as in the present case) embolization cannot effectively reduce the size of lesion and surgery is the only effective treatment.

## CONFLICT OF INTEREST

The authors declare no conflict of interest.
